# Selection and Characterization of DNA Aptamers for Egg White Lysozyme

**DOI:** 10.3390/molecules15031127

**Published:** 2010-03-02

**Authors:** Dinh T. Tran, Kris P. F. Janssen, Jeroen Pollet, Elke Lammertyn, Jozef Anné, Ann Van Schepdael, Jeroen Lammertyn

**Affiliations:** 1 BIOSYST-MeBioS, Katholieke Universiteit Leuven, Willem de Croylaan 42, B-3001 Leuven, Belgium; E-Mails: ThiDinh.Tran@biw.kuleuven.be (D.T.T.); Kris.Janssen@biw.kuleuven.be (K.P.F.J.); Jeroen.Pollet@biw.kuleuven.be (J.P.); 2 Laboratory of Bacteriology, Rega Institute, Katholieke Universiteit Leuven, Minderbroedersstraat 10, B-3000 Leuven, Belgium; E-Mails: Elke.Lammertyn@lrd.kuleuven.be (E.L.); Jozef.Anne@rega.kuleuven.be (J.A.); 3 Laboratory for Pharmaceutical Analysis, O&N II Herestraat 49 - bus 923, Katholieke Universiteit Leuven, B-3000 Leuven, Belgium; E-Mail: Ann.VanSchepdael@pharm.kuleuven.be (A.V.S.)

**Keywords:** aptamer, capillary electrophoresis, food allergen, lysozyme, systematic evolution of ligands by exponential enrichment

## Abstract

We have selected aptamers binding to lysozyme from a DNA library using capillary electrophoresis-systematic evolution of ligands by exponential enrichment. During the selection process the dissociation constant of the ssDNA pool decreased from the micromolar to the low nanomolar range within five rounds of selection. The final aptamer had a dissociation constant of 2.8 ± 0.3 nM, 6.1 ± 0.5 nM, and 52.9 ± 9.1 nM as determined by fluorescence anisotropy, surface plasmon resonance and affinity capillary electrophoresis respectively. The aptamers were successfully challenged for specificity against other egg white proteins. The high affinity aptamers open up possibilities for the development of aptamer based food and medical diagnostics.

## 1. Introduction 

Lysozyme from hen egg white has been used not only as an anti-inflammatory drug in the treatment of wounds and infections but also as a natural antibacterial agent in food preservation due to its muraminidase activity [1]. Commercially, lysozyme is most often used in cheese production for the prevention of late gas blowing that is caused by the growth of *Clostridium tyrobutyricum* [2,3]. In the field of enology, it is used to prevent the color loss of red wine [4] and reduce the lactic bacteria flora in musts and wines after completion of malolactic fermentation [5]. Lysozyme has also been added in surimi products, shrimp, and sausage to prolong the shelf-life of these products [6,7,8]. 

Besides its useful properties, lysozyme also has its drawbacks. Clinical studies revealed that lysozyme is a dominant egg-white allergen named Gal d 4 [9]. Lysozyme can cause allergic reactions even when present in minute amounts [10,11,12]. Hence it is of importance to design highly sensitive bioreceptors for detection of this substance in products intended for consumption by allergic patients.

Currently, enzyme-linked immunosorbent assay (ELISA) using antibody for quantification of lysozyme is commercially available. The minimal sensitivity of this assay is 0.021 ng/mL (Calbiotech Inc, Spring Valley, CA, USA). However, the common disadvantages of ELISA are the complicated succession of steps, long reaction time and the expensive reagents involved. 

In recent years, there is an increase in the use of aptamers as biorecognition molecules for biosensors. As bioreceptors, aptamers have distinct advantages over antibodies, such as elimination of batch to batch variability, low cost of manufacturing, and resistance to denaturation and degradation [13]. Aptamers are selected from synthetic nucleic acid libraries using the iterative SELEX (Systematic Evolution of Ligands by EXponential enrichment) process for a wide range of targets such as proteins [14,15,16], peptides [17], amino acid [18], and organic dyes [19]. There have been a number of reviews describing SELEX in detail [20,21,22]. Cox and Ellington [23] have selected aptamers for lysozyme using automated selection. Briefly a typical round of selection involved in incubation of the RNA pool with lysozyme captured on magnetic beads, filtration to separate bound sequences from unbound species, and amplification to generate an enriched RNA pool for the next round. After the final round, they obtained an aptamer with a dissociation constant (*K_d_*) of 31 nM. This aptamer was further optimized by Kirby *et al*. [24] to accommodate both immobilization and sensing. Subsequently, the modified aptamer was used as a receptor molecule to develop biosensors for lysozyme detection based on different transduction mechanisms [25,26,27,28,29,30]. 

In this paper, we present the selection of DNA aptamers for lysozyme with high affinity and selectivity using capillary electrophoresis-SELEX (CE-SELEX). The main advantages of CE-SELEX over the conventional SELEX are that this method provides very strong power of separation, selections are carried out in solution, thus reducing the non-specific binding introduced by stationary support and it is possible to monitor the evolution of the overall affinity increase of selected aptamers online [31]. 

The objective of this study was to select DNA aptamers for lysozyme with high affinity and selectivity using CE-SELEX. The selected aptamers were characterized for binding affinity using fluorescence anisotropy, affinity capillary electrophoresis and surface plasmon resonance and finally challenged with other egg white proteins to assess specificity. The selected aptamers with nanomolar dissociation constants may function as biorecognition molecules to develop biosensors for the detection and quantification of lysozyme in food and pharmaceuticals to ensure compliance with allergen labeling and improve consumer protection.

## 2. Results and Discussion 

### 2.1. Selection of aptamers

**Figure 1:**
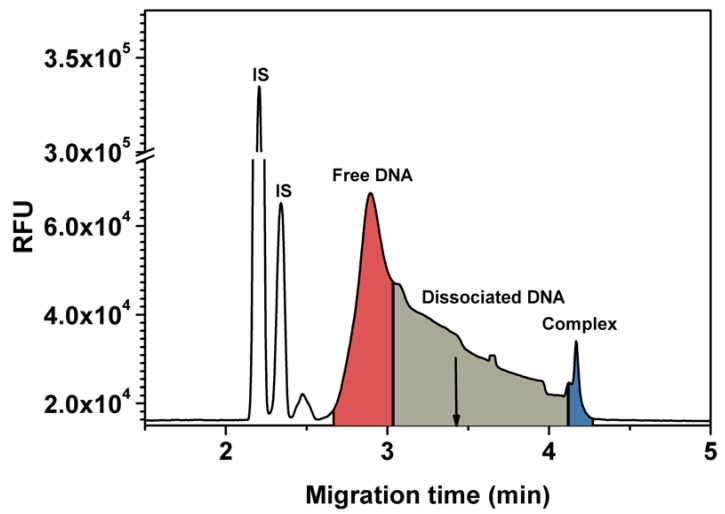
Choosing an aptamer collection window. The injection mixture contained 20 nM fluorescently labeled DNA and 1600 nM lysozyme. CE conditions: TGK buffer, pH = 8.3; 1psi/5s injection; 25 ºC; LIF detection; 40.2-cm, 50-µm-i.d. capillary; 20-kV separation voltage. 50 nM BCECF was used as internal standard (IS). The cutoff point where collection of binding sequences is started is marked on the electropherogram by the arrow.

CE-SELEX was used to select aptamers against egg-white lysozyme. In order to determine the aptamer collection window, the ssDNA library was incubated with the target. After equilibrium was reached, the mixture was injected into the capillary. Since the electrophoresis mobility of a solute in the capillary depends linearly on its charge to mass ratio, the complex migrated slower than the free DNA when reverse polarity is applied over the capillary. Figure 1 shows that the free DNA reached the detector after 3 min while the peak of the complex appeared after 4.2 min. To avoid contamination by unbound DNA during sample collection, the collection window was chosen after 3.4 min of separation. 

**Figure 2:**
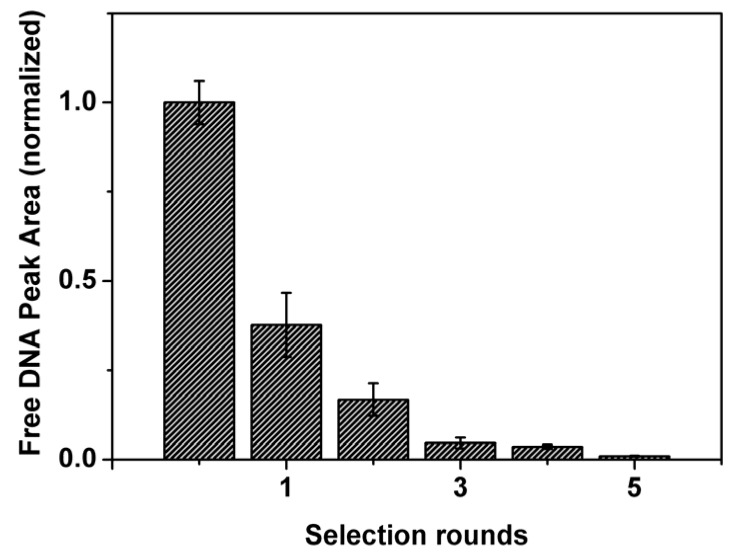
Affinity of the DNA library (first bar) and the enriched DNA pools to lysozyme after each CE-SELEX round. Free DNA peak areas were measured by affinity capillary electrophoresis. Error bars indicate the 95% confidence intervals as determined from non-linear least-squares regression analysis.

The improvement in binding of the DNA pool with lysozyme after each round of selection was initially evaluated using affinity capillary electrophoresis (ACE). The results in Figure 2 reveal that the average affinity of the DNA pool for the target increased significantly in the first three rounds of selection. This is represented by a decrease in free DNA. In round 5, the estimated affinity leveled off, at least two orders of magnitude below the bulk affinity of the naive DNA library. In total, the selection of aptamers for lysozyme required only five selection rounds. This clearly shows the advantages CE-SELEX can hold over traditional SELEX which normally requires anywhere between eight to 15 rounds to obtain aptamers with dissociation constant values in the nanomolar range [32]. 

Sequence alignment using the ClustalX software program revealed that there were five clones remaining in the enriched DNA pool obtained after the final round. Their sequences are given in Table 1. None of these aptamers has a sequence region which is identical to that of aptamers selected for lysozyme in the study conducted by Cox and Ellington [23] using automated selection. Three clones namely Apta1, Apta3 and Apta8 were selected, based on their abundance, and synthesized for further characterization with respect to affinity and specificity.

### 2.2. Evaluation of the aptamer binding affinity

**Figure 3:**
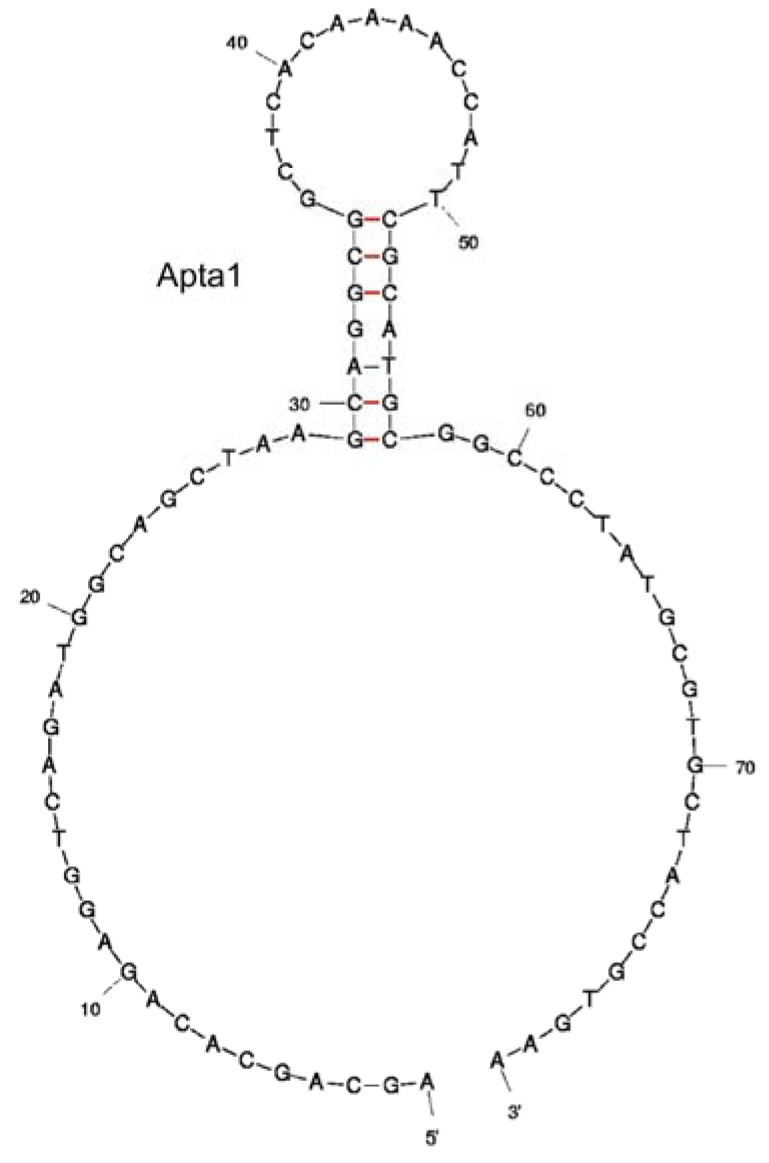
Secondary structure prediction of Apta1. The structure was determined using the *mfold* tool.

The binding affinity of the aptamers was firstly determined using ACE. All three clones showed high affinity to lysozyme and had very similar *K_d_* values when measured using ACE (52.9 ± 9.1 nM, 47.1 ± 16.3 nM, 52.1 ± 12.7 nM for Apta1, Apta3 and Apta8 respectively). This illustrates that clones with different sequences in the final pool have a comparable affinity for lysozyme. Analyses of the secondary structure of the three aptamers using the *mfold* program showed that the primer regions of Apta1 were in an exterior loop (Figure 3) while the reverse primer of Apta3 was arranged in the stem-loop structure. The free energy of Apta1 was also significantly lower than that of Apta3 and Apta8. Therefore, only Apta1 was used in fluorescence anisotropy (FA) and surface plasmon resonance (SPR) for binding study.

**Figure 4:**
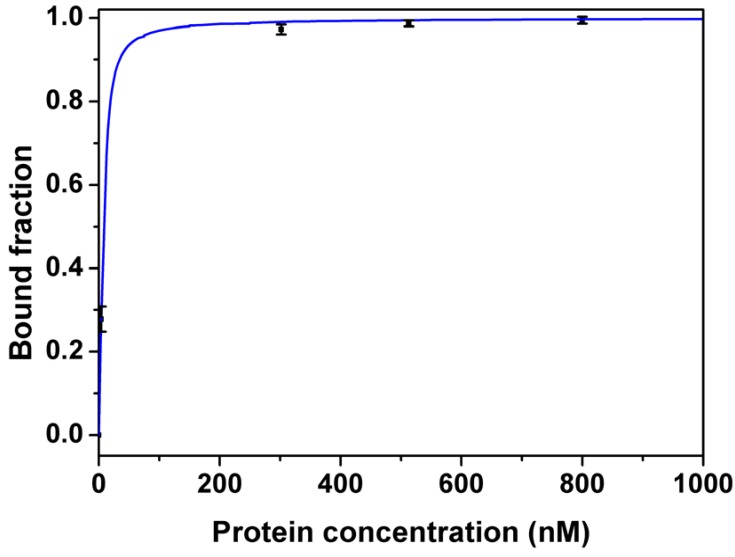
Binding curves of aptamer (Apta1) to lysozyme measured by fluorescence anisotropy. 60 µL of 20 nM ssDNA was incubated with the same volume of different concentrations of lysozyme (0, 3.8, 302, 513 and 800 nM) in quartz cuvettes. The dissociation constant (*K_d_*) was estimated using non-linear least-squares regression analysis. Full line represents the fitted data and symbols represent the measured data given as averages of three measurements. Error bars indicate the 95% confidence intervals.

Figure 4 shows the binding curve of Apta1 to lysozyme measured by FA. The *K_d_* for Apta1 was estimated to be 2.8 ± 0.3 nM. This *K_d_* value is about 17 times lower than that obtained using ACE. This is explained by the fact that under the high voltage applied during CE separation, the equilibrium of the mixture was no longer maintained. As a consequence, the complex constantly dissociated which caused smearing of the unbound DNA region (grey area in Figure 1). A part of the complex, which was still intact, generated a single peak (blue area). The decay of the complex made it difficult to determine the end point of the free DNA peak when integrating. In our study, the peak area of the free DNA is shown in the red region in Figure 1. The *K_d_* value obtained by ACE was higher than that obtained by FA which means that we integrated not only the peak of free DNA but also a part of DNA which dissociated from the complex. So, FA is a more accurate technique to determine the affinity of the aptamer to lysozyme.

**Figure 5:**
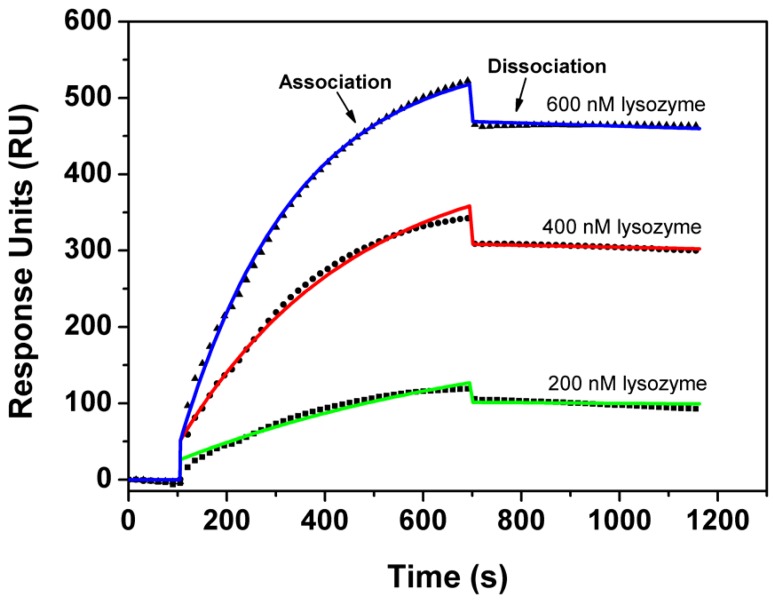
Global association/dissociation analysis of the kinetic SPR data on the binding interaction of lysozyme and Apta1. Biotinylated aptamers (0.5 µM) were immobilized on a sensor chips and the protein solutions (200, 400, 600 nM) were injected over the surface.

In the SPR measurement, Apta1 was biotinylated and immobilized onto a streptavidin coated SPR-chip. In order to minimize mass diffusion effects, the density of the receptor layer was kept to 700 pg of aptamers per mm^2^ (700 RU). Figure 5 illustrates the high correspondence of the model to the measured kinetic data. Based on this model we estimated the association rate constant, *k_on_* = 6.99×10^3^ ± 0.04×10^3^ M^-1^s^-1^ and the dissociation rate constant, *k_off_* = 4.3×10^-5^ ± 0.3 × 10^-1^ s^-1^, and hence a *K_d_* of 6.1 ± 0.5 nM, confirming the results of the affinity study with fluorescence anisotropy.

However, some caution should be taken when interpreting these SPR results, since we observed limited non-specific interactions between the lysozyme and the bare streptavidin chip. This might be a result of electrostatic interactions between the protein and the sensor surface and hydrophobic interactions between the streptavidin and the lysozyme. As reported previously, these electrostatic interactions can also play an important role in the formation of the complex by a of ‘linger and lock’ mechanism [33]. We conclude that although the value of the *K_d_* is very similar to the results obtained in the FA experiments, the kinetic behavior of an lysozyme aptamer bound at a surface might be slightly different compared to the behavior of an unbound aptamer in solution.

### 2.3. Evaluation of binding specificity

**Figure 6:**
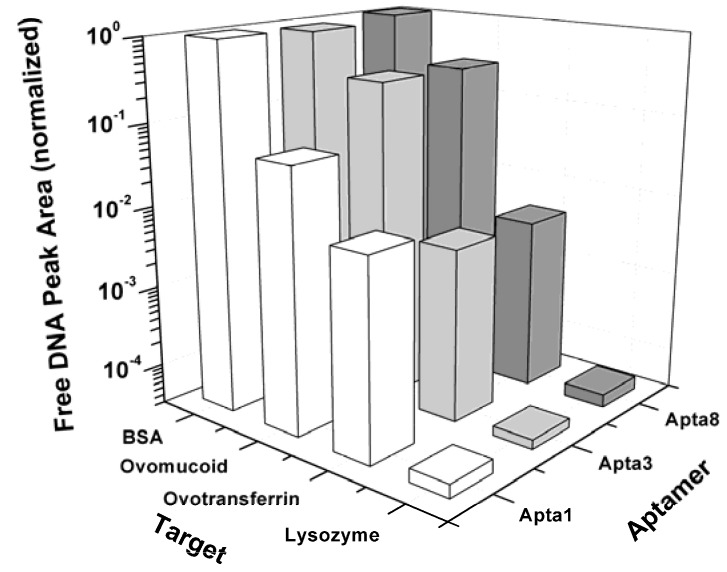
Specificity of the selected aptamers Apta1, Apta3 and Apta8 for lysozyme and for ovomucoid, ovotransferrin and BSA measured with affinity capillary electrophoresis.

In order to examine the specificity of the selected aptamers to lysozyme, the binding assays were performed using ACE with ovomucoid and ovotransferrin, two proteins causing allergic reactions in egg white, and BSA which is normally used to block the non-specific binding of aptamers to a transducer platform in biosensor applications. Figure 6 indicates that the affinity of the clones Apta1, Apta3 and Apta8 for ovomucoid is orders of magnitude lower than for lysozyme. Although a very small amount of complex was found when checking the binding affinity of the clones to ovomucoid, care should be taken when interpreting these results. According to Ebbehoj *et al.* [34] commercial ovomucoid is often contaminated with lysozyme, hence, the bound fraction observed might be due to the binding of DNA to lysozyme but not to ovomucoid. For BSA, the affinities of the three clones were determined to be about 5 orders of magnitude lower than the affinity values for lysozyme. These results illustrate that the selected aptamers not only bind strongly to lysozyme but are also very specific and do not bind to other egg white allergenic proteins. 

## 3. Experimental 

### 3.1. Reagents

Trizma base, lysozyme, ovomucoid and ovotransferrin from chicken egg white, were purchased from Sigma-Aldrich (St. Louis, MO, USA), potassium phosphate dibasic, glycine, sodium hydroxide and EDTA were purchased from ACROS (Fair Lawn, NJ, USA). 2',7'-bis-(2-carboxyethyl)-5-(and-6)-carboxyfluorescein (BCECF) was purchased from Invitrogen (Paisley, UK). ‘Small fragments’ agarose, the DNA library, PCR primers and PCR reagents were purchased from Eurogentec (Liège, Belgium) except for TAQ polymerase and PCR reaction buffer that were purchased from Sphaero-q (Gorinchem, The Netherlands). The DNA library contained 20 nucleotides in the primer regions and 40 nucleotides in a central randomized region (5’-AGC AGC ACA GAG GTC AGA TG - N40 - CCT ATG CGT GCT ACC GTG AA-3’). A 6-carboxyfluorescein (6-FAM) was chemically attached to the 5’ end of a DNA sequence. The electrophoresis buffer consisted of 25 mM trizma base, 192 mM glycine, and 5 mM K_2_HPO_4_ (TGK) at a pH of 8.3. Buffer solutions were prepared using HPLC grade water (VWR, Fontenay sous Bois, France) and filtered through a 0.22 µm filter (Millipore, Bedford, MA, USA). 

### 3.2. Capillary electrophoresis operational parameters

All CE experiments were performed using a P/ACE MDQ capillary electrophoresis system combined with a laser-induced fluorescence (LIF) detector (Beckman Coulter, Inc., Fullerton, CA, USA). As an excitation source a 3 mW argon ion laser with a wavelength of 488 nm was used and emission was monitored using a 520/10 nm band pass filter. For all separations a poly(vinyl alcohol) (PVA)-coated capillary (Beckman Coulter, Inc.) with 50 µm inner diameter, 360 µm outer diameter was used. The total capillary length was 40.2 cm with a length to detector of 30 cm. The coating of the inner wall is intended to decrease non-specific binding of the target to the capillary wall. At the beginning of each experiment, the capillary was rinsed with de-ionized water for 30 min, followed by a rinse with electrophoresis buffer for an additional 15 min. Between injections, the column was rinsed with de-ionized water for 3 min and with running buffer for 2 min. The temperature of the capillary was maintained at 25 ºC for all separation experiments. Electrophoresis data were collected and analyzed using a 32Karat software, version 8.0 (Beckman Coulter, Inc.).

### 3.3. Aptamer selection protocol 

The DNA library and the amplified ssDNA pool after each round of selection were dissolved in TGK electrophoresis buffer, heated to 75 ºC for 10 min and allowed to cool down on ice. The heating and cooling steps are intended to minimize any misfolding of the aptamers prior to incubation with the protein target. In the first round of selection, 5 µL of a 500 µM solution of the DNA pool was mixed with 5 µL of 200 nM lysozyme and the mixture was incubated for 30 min at room temperature before electrophoretic separation. In the following rounds, the enriched DNA pools were incubated with decreasing lysozyme concentrations; 50 nM in round 2, 25 nM, in round 3, 12.5 nM in round 4 and round 5 under conditions identical to those described above. This was done to increase the stringency of the overall selection process.

After incubation, a plug of equilibrium mixture was hydrodynamically injected into the capillary by applying a pressure of 5 psi for 10 s, which corresponds to an injection volume of approximately 130 nL. Separation was carried out at a constant voltage of 20 kV for 4.5 min with reverse polarity. During this time any unbound species were allowed to elute from the column into a vial of run buffer that was later discarded. When the complexed species reached the end of the column, the target vial was switched and the binding sequences remaining on the capillary were flushed into a collection vial containing 30 µL of water by pressure rinsing of the column of 10 psi for 1 min. The exact time at which the complex reaches the end of the capillary was calculated by correcting the complex detection time for the distance between the detection window and the capillary end according to the factor $f=L_{total}/L_{\det ector}$. Two repeated separations were carried out for each selection round to increase the number of collected sequences.

### 3.4. PCR and dsDNA separation

After separation by CE, the collected volume containing active sequence was amplified using the polymerase chain reaction (PCR) protocol described by Mendonsa and Bowser [35]. Briefly, a 30 µL volume of the collected solution was divided into 6 volumes of 5 µL each. PCR master mix was added to give a final volume of 50 µL. All vials were then transferred into a thermal cycler (PerkinElmer, Massachusetts, USA) and heated to 95 ºC for 5 min to denature the DNA-lysozyme complex. Then the PCR cycling was started. A total of 18 cycles was performed in this way. The size and purity of PCR products was subsequently checked by running samples in 2.5% agarose gel for 30 min at 140V followed by ethidium bromide-staining. 

After amplification, the product was purified using PCR clean-up kit (NucleoSpin, Düren, Germany) to remove any residual primers. The obtained dsDNA sequences were then separated into single stranded form using streptavidin functionalized magnetic beads (Invitrogen, Oslo, Norway). A 20 µL suspension of the beads was washed three times with 100 µL single concentration binding buffer (10 mM trizma base, 50 mM NaCl, and 1 mM EDTA pH = 7.5). The cleaned beads were then re-suspended in 50 µL double concentration binding buffer. Fifty µL of purified dsDNA was added in the mixture and was allowed to react at room temperature for 1 hour under gentle shaking to ensure all dsDNA was properly bound to the magnetic beads. The beads were then washed three times with 100 µL single concentration binding buffer. The beads were re-suspended once more in 15 µL of 0.2 M NaOH and left for 5 min under continuous shaking at room temperature to release the desired ssDNA from its complement that remained bound to the magnetic beads which were removed from the solution. Finally, the ssDNA was precipitated by bringing 15 µL DNA solution into a microcentrifuge tube containing 45 µL absolute ethanol and 1.5 µL of 3M CH_3_COONa at pH 5.5. Samples were incubated at −20 ºC overnight. The precipitated ssDNA was separated from the supernatant by centrifugation at 20.000 g for 60 min at 4 ºC. Subsequently, the supernatant was decanted from the sample tube. The ssDNA pellet was allowed to dry at 65 ºC for 5 min. The dried pellet was then re-suspended in electrophoresis buffer to serve as a new enriched ssDNA pool for further selection rounds.

### 3.5. Cloning and sequencing

The DNA pool obtained after the fifth round of selection was subjected to PCR amplification with non-labeled primers. The conditions were modified to accommodate further cloning and sequencing of the obtained DNA pool. The PCR products were further purified using a gel extraction kit (NucleoSpin, Düren, Germany). The eluted ssDNA was cloned into the pGEM-T easy vectors (Promega, Madison, WI, USA). High efficiency, *E. coli* competent cells (≥1 × 10^8^cfu/µg DNA) (Promega, Madison, USA) were transformed using the vectors containing DNA fragments of interest. The successful transformation was verified by PCR of the resulting bacterial colonies using non-labeled primers to amplify the inserts and followed by electrophoresis on a 2.5% agarose gel. Fourteen colonies were then chosen at random for sequencing using the M13 forward primer (Genetic Service Facility, University of Antwerp, Belgium). The secondary structure of the selected aptamers was analyzed using *mfold* program [36].

### 3.6. Binding affinity 

#### 3.6.1. Affinity capillary electrophoresis

In order to check the progress of aptamer selection and to assess the binding affinity of the aptamer pool, an average affinity value—Expressed as peak area of free DNA—was determined after every round of selection with CE. For this purpose, a 20 nM sample of ssDNA was pre-incubated with increasing amounts of lysozyme in the presence of BCECF as internal standard (IS). After 30 min of incubation at room temperature the mixture was separated electrophoretically under conditions identical to those used for aptamer selection except that the samples were injected under the pressure of 1 psi for 5s. The peak areas of the relevant species on the resulting electropherograms were determined by integration. The model presented by Tetin and Hazlett [37] was applied to determine the *K_d_* of aptamers using the non-linear least-squares regression analysis performed with statistical analysis system (SAS) (SAS Institute, Inc., Cary, NC, USA).

#### 3.6.2. Fluorescence anisotropy

FA measurements were performed using a SPEX FluoroLog 3 Fluorometer (Horiba Jobin Yvon Inc., Edison, NJ, USA). In this assay, 60 µL of 20 nM ssDNA was incubated with the same volume of different concentrations of lysozyme (0, 3.8, 302, 513 and 800 nM) in quartz cuvettes (Hellma, Germany). The fluorescein-labeled conjugates were excited at 472 nm and fluorescence was collected at 512 nm. The anisotropy values were used to estimate *K_d_* using the same model structure as applied for ACE.

#### 3.6.3. Surface plasmon resonance

SPR experiments were performed on a Biacore 3000 (Biacore, GE Healthcare, Uppsala, Sweden). Biotinylated aptamers (0.5 µM in 10 mM Tris-HCl, 300 mM NaCl, and 1 mM EDTA at pH = 7.5) were immobilized on streptavidin coated SA-sensor chips (Biacore). The validated aptamers were extended with 24 thymidine bases at the 3’ biotin binding site, to give them maximum flexibility for binding with the target [38]. Conforming to the ACE and the FA experiments, the kinetic interaction between the aptamer and lysozyme was studied in TGK buffer. The regeneration of the aptamer coated surface was achieved with a 30 s pulse of a 10 mM glycine/HCl buffer (pH = 2.5). The statistical analysis of the kinetic data of the SPR measurements was done based on a 1:1 Langmuir model using Prism (Graphpad Software, San Diego, CA, USA).

## 4. Conclusions

We have successfully selected DNA aptamers with a strong affinity and selectivity towards lysozyme using capillary electrophoresis. This technique allows monitoring of the relative affinity increase of the aptamer pool during the SELEX procedure, but is less accurate for the absolute estimation of the dissociation constant due to a poor peak resolution. Following selection the affinity constants for the complexation reaction of selected aptamers with lysozyme were accurately determined using fluorescence anisotropy and resulted in a value of 2.8 nM. SPR measurements confirmed these results but also illustrated the tendency of lysozyme for non-specific binding to the sensor surface. The selection and characterization of this high affinity aptamer opens up possibilities for the development of aptamer based food and medical diagnostics. 

## Figures and Tables

**Table 1:** Sequences of the clones remaining after five rounds of selection using CE-SELEX. Only the random region (40 nucleotides) is given.

Clone ID	Sequence of random region (5’ to 3’)
Apta1	GCAGCTAAGCAGGCGGCTCACAAAACCATTCGCATGCGGC
Apta2	GCGTGGGCAGCTAGCACCGATGGTTCTATCGTGGGCTCCG
Apta3	GCGGGTCGGTTGCTCGCTTCGCCCGATCGGTCTAAGGGTG
Apta4	GCGCAAGGTCATCGCATCGCGTCGGAATGGGCTACAGGTG
Apta8	GCACCTTGATGACATGATAGTCGTTGTGTATGCAGTTGGC
